# Ubiquitylation of Terminal Deoxynucleotidyltransferase Inhibits Its Activity

**DOI:** 10.1371/journal.pone.0039511

**Published:** 2012-07-11

**Authors:** So Maezawa, Rie Fukushima, Toyofumi Matsushita, Tomoyoshi Kato, Yoshiki Takagaki, Yoshihiro Nishiyama, Sachiko Ando, Takuro Matsumoto, Kousuke Kouda, Takahide Hayano, Masahiro Suzuki, Kotaro Koiwai, Osamu Koiwai

**Affiliations:** Department of Applied Biological Science, Faculty of Science and Technology, Tokyo University of Science, Noda, Chiba, Japan; Institute of Enzymology of the Hungarian Academy of Science, Hungary

## Abstract

Terminal deoxynucleotidyltransferase (TdT), which template-independently synthesizes DNA during V(D)J recombination in lymphoid cells, is ubiquitylated by a BPOZ-2/Cul3 complex, as the ubiquitin ligase, and then degraded by the 26 S proteasome. We show here that TdT is ubiquitylated by the Cul3-based ubiquitylation system *in vitro*. Because TdT could also be ubiquitylated in the absence of Cul/BPOZ-2, we determined that it could also be directly ubiquitylated by the E2 proteins UbcH5a/b/c and UbcH6, E3-independently. Furthermore, the ubiquitylated TdT inhibited its nucleotidyltransferase activity.

## Introduction

Terminal deoxynucleotidyltransferase (TdT) is a unique DNA polymerase that catalyzes DNA polymerization in the absence of a DNA template [1]. Genetic rearrangement of immunoglobulin (Ig) and T-cell receptor (TcR) genes enhances their diversity, and extra nucleotides (N region) are inserted during rearrangement at the V-J, V-D, and D-J junctions by TdT [2], [3]. After synthesis of the N region, TdT in pro-B- or pro- and pre-T-cells rapidly disappears. It may be degraded by ubiquitylation in lymphoid cells [4].

The ubiquitylation system is an essential regulator of multiple cellular processes that marks proteins for proteasome-mediated degradation, receptor internalization, endocytic trafficking [5], [6], histone modification [7], vesicular trafficking [8], DNA repair [9], [10], viral budding [11], [12], or transcriptional regulation [13], [14]. Ubiquitin (Ub), a 76-aa polypeptide, is attached to the ε-amino group of lysine residue in target proteins by multienzymes. A Ub-activating enzyme (E1) initially activates Ub ATP-dependently. The activated Ub is then transferred to the cysteine in the active site of a Ub-conjugating enzyme (E2) by a transesterification reaction. E2s are grouped into four classes (class I, II, III and IV) that contain, respectively, a catalytic core domain (UBC) composed of ∼150 aa, a UBC and a COOH-terminal extension, a UBC and an NH_2_-terminal extension, and a UBC and both of NH_2_- and COOH-terminal extensions. The third component, a Ub protein ligase (E3), cooperates with E2 to transfer Ub to the substrate.

E3s are grouped into two classes [15]. One class, RING finger proteins, directly transfers Ub from E2 to substrates. E3s in the second class (homologous to E6-AP COOH terminus [HECT] domain proteins) contain a cysteine in the active site; these enzymes first accept Ub from E2 and then transfer it to the substrate. After transfer of the first Ub to a lysine in the target protein, subsequent Ubs are added sequentially. When the Ub polymer is synthesized using K48 of Ub to attach each component, the poly-Ub functions to signal delivery of the protein to the 26 S proteasome for degradation. When the Ub components are polymerized on other Ub lysines (e.g., K63), the target protein is not degraded. Mono-ubiquitylation of a protein can also serve as a marker for DNA repair [16].

Protein ubiquitylation is balanced by a set of de-ubiquitylating isopeptidases that release Ub from ubiquitylated proteins [17]. Protein ubiquitylation in eukaryotes is regulated by several E2s and E3s. In humans, more than thirty E2s and hundreds of putative E3 ligases have been identified. In addition, different E2s can interact with a common E3, and a single E2 can function together with a variety of E3s including the RING finger and the HECT domain types [18], [19], [20].

We recently reported that BPOZ-2 functions as a substrate-specific adaptor for Cul3-based E3 ligase and that TdT is a substrate of the BPOZ-2/Cul3 complex [4]. BPOZ-2 is a human counterpart of yeast Btb3p and is involved in the growth-suppressive effect of tumor suppressor phosphatase and tensin homologue deleted on chromosome 10 (PTEN), which dephosphorylates phosphatidylinositol 3,4,5-triphosphate (PIP3) and antagonizes phosphoinositide 3-kinase (PI3K)-dependent growth signaling, resulting in an inhibition of protein synthesis and cell growth or promotion of apoptosis [21]. Cullin-RING ligases (CRLs), including Cul3, operate as multi-subunit complexes composed of a cullin, a RING H2 finger protein, and a substrate-binding moiety. Cul3 connects the RING H2 finger protein Ring-box (Rbx) 1 or 2 and a substrate-binding subunit containing a BTB (Bric-a-brac, Tramtrack, Broad-complex) domain [22]. BPOZ-2 contains two BTB domains, through which BPOZ-2 binds to Cul3.

Here, we reconstituted the BPOZ-2/Cul3-mediated TdT ubiquitylation system *in vitro*, using purified proteins. TdT could be ubiquitylated in the absence of the BPOZ-2/Cul3 complex. Furthermore, the ubiquitylated TdT retained its DNA binding ability but reduced its nucleotidyltransferase activity.

## Results

### TdT is Ubiquitylated *in vitro*

**Figure 1:**
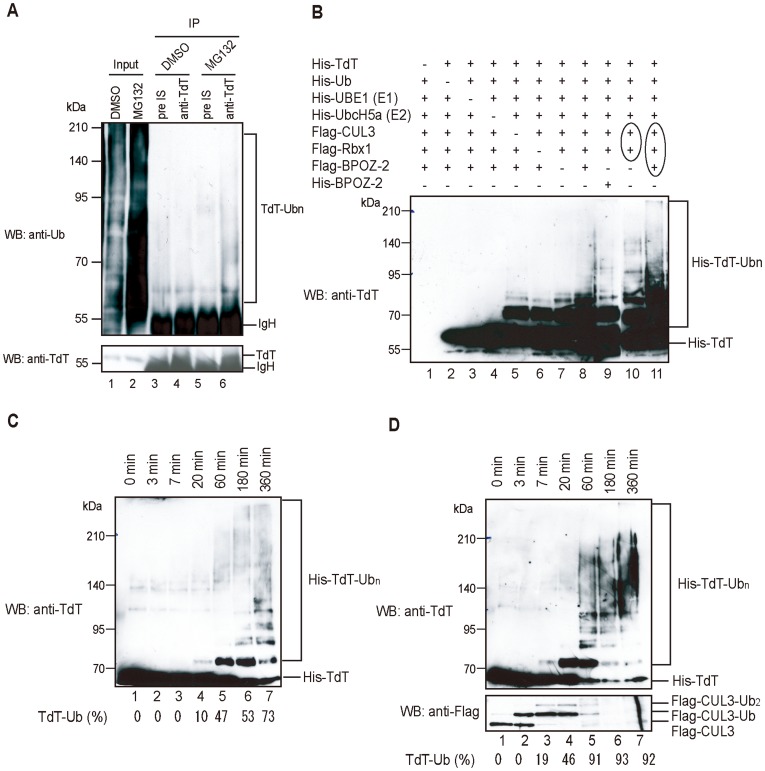
TdT ubiquitylation *in vivo* or *in vitro*. (A) Endogenous TdT ubiquitylation in Jurkat cells. Immunoprecipitation was carried out using an anti-TdT antibody (lanes 4 and 6) or rabbit pre-immune serum (pre-IS) (lanes 3 and 5). Immunoprecipitants were subjected to SDS-PAGE and immunoblotted with anti-Ub (FK2) or anti-TdT antibody. One mg of whole cell lysate was used for each immunoprecipitation. (B) *In vitro* TdT ubiquitylation. The substrate His-TdT was incubated under ubiquitylation conditions with purified proteins. His-TdT and His-BPOZ-2 were purified from *E. coli* and the Flag-tagged proteins were purified from 293 T cells. To obtain Cul3/Rbx1 or BPOZ-2/Cul3/Rbx1 complexes, 293 T cells were co-transfected with the circled plasmids (lanes 10 and 11). After electrophoresis with a denaturing 7.5% polyacrylamide gel, ubiquitylated TdT was detected using an anti-TdT antibody. (C and D) Kinetics of TdT ubiquitylation in the absence (C) or presence (D) of the BPOZ-2/Cul3/Rbx1 complex. The incubation time after the addition of His-TdT is given above. The reaction products were separated by 7.5% SDS-PAGE and immunoblots were probed with anti-TdT antibody or anti-Flag antibody. The ratio of ubiquitylated His-TdT to unmodified His-TdT was determined with ImageJ. The band density for ubiquitylated His-TdT was defined as 100%.

Over-expressed TdT in 293 T cells is ubiquitylated by BPOZ-2/Cul3-based ubiquitylation and degraded by the 26 S proteasome system [4]. To investigate whether TdT is ubiquitylated *in vivo*, we first attempted to detect ubiquitylated TdT in Jurkat cells by Western blotting, using an anti-TdT antibody. We were able to detect the ubiquitylated TdT in lysates of Jurkat cells treated with MG132 (Figure 1A).

To confirm TdT ubiquitylation *in vitro*, we purified E3 from 293 T cells over-expressing Flag-Cul3, Flag-Rbx1, Flag-BPOZ-2. We then ubiquitylated TdT using recombinant E1 and E2 (UbcH5a), purified from lysates of *E. coli*, and E3. The association between Rbx1 or BPOZ-2 and Cul3 was confirmed by co-immunoprecipitation using an anti-BPOZ-2 antibody (data not shown). Incubating the BPOZ-2/Cul3/Rbx1 complex with purified TdT resulted in the formation of characteristic incremental ubiquitin ladders (Figure 1B, lanes 8). However, when His-BPOZ-2 expressed in *E. coli* was used instead of Flag-BPOZ-2 expressed in 293 T cells, no notable increase in E3 activity was detected (lane 9). These results suggest that BPOZ-2 is modified in 293 T cells to function as an adaptor for Cul3. When purified Cul3/Rbx1 or BPOZ-2/Cul3/Rbx1 complex was used for TdT ubiquitylation, high E3 activities were detected (lanes 10 and 11), indicating that the Cul3/Rbx1 or BPOZ-2/Cul3/Rbx1 in 293 T cells critically enhances E3 activity. Surprisingly, however, TdT was ubiquitylated even in the absence of Cul3 (lane 5).

We re-examined whether TdT is E3-independently ubiquitylated *in vitro* by Western blotting. As shown in Figure 1C, TdT was ubiquitylated even when E3 was not added to the *in vitro* ubiquitylation system. We also examined the effect of BPOZ-2/Cul3/Rbx1 complex on TdT ubiquitylation. As shown in Figure 1D, the BPOZ-2/Cul3/Rbx1 complex enhanced TdT ubiquitylation.

### TdT Directly Binds to E2

**Figure 2:**
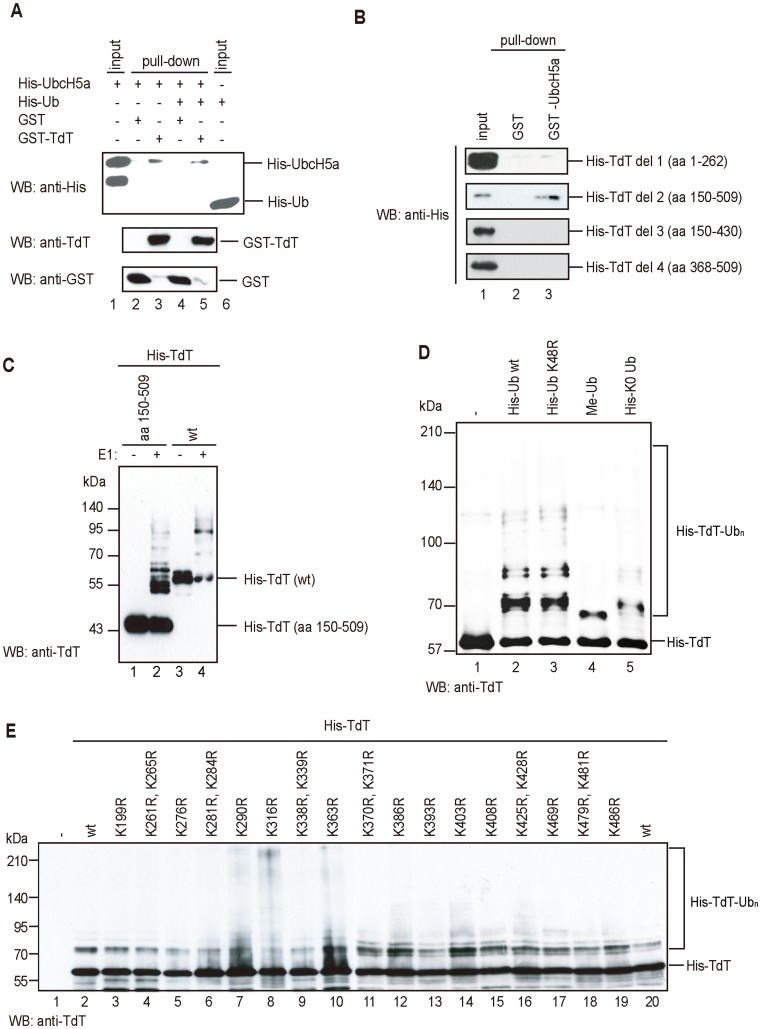
TdT directly binds to E2. (A) UbcH5a (E2) binds to TdT *in vitro*. His-UbcH5a was incubated with GST-bound (lanes 2 and 4) or GST-TdT-bound (lanes 3 and 5) Glutathione Sepharose 4B in the absence (lanes 2 and 3) or presence (lanes 4 and 5) of His-Ub. Proteins bound to the beads were eluted by boiling with Laemmli buffer. The eluates were subjected to SDS-PAGE and analyzed by immunoblotting with anti-His, anti-TdT, or anti-GST antibody. (B) The pol β-like region in TdT binds to UbcH5a. His-TdT deletion mutants were incubated with GST-bound (lane 2) or GST-UbcH5a-bound (lane 3) Glutathione Sepharose 4B. Proteins bound to the beads were eluted with Laemmli buffer after boiling. The eluates were subjected to SDS-PAGE and analyzed by immunoblotting using an anti-His antibody. (C) E3-independent ubiquitylation of either His-TdT (aa 150–509 or wt) was carried out with UBE1, UbcH5a, and Ub. Ubiquitylated proteins were detected by immunoblotting using an anti-TdT antibody. (D) TdT is poly-ubiquitylated through lysine(s) other than Lys48. TdT was ubiquitylated in a reaction mixture without Ub (lane 1) or containing UbcH5a with His-Ub wt (lane 2), His-Ub K48R (lane 3), Me-Ub (lane 4), or lysine-less (K0) Ub (lane 5). Ubiquitylated His-TdT was detected by immunoblotting using an anti-TdT antibody. (E) *In vitro* ubiquitylation of TdT mutants. E3-independent ubiquitylation was carried out for wild-type (wt) or point-mutated His-TdTs. TdT was ubiquitylated in the reaction mixture containing UBE1, UbcH5a, and Ub. His-TdT ubiquitylation was detected by immunoblotting using an anti-TdT antibody.

Since TdT was ubiquitylated by E2 in the absence of E3, we then addressed the question of direct binding between TdT and E2 using a GST pull-down assay, with GST-TdT as the bait and His-UbcH5a, which catalyzes TdT ubiquitylation, or His-Ub as the prey. As shown in Figure 2A, GST-TdT bound to UbcH5a in the presence or absence of His-Ub and did not bind to Ub, indicating that TdT directly binds to E2.

To identify the UbcH5a binding region in TdT, we constructed four TdT deletion mutants and performed GST pull-down assays using UbcH5a as the bait. As shown in Figure 2B, the N-terminal region of TdT (del 1; 1–262), including the BRCT domain which is involved in protein-protein interactions, did not bind to UbcH5a, whereas the C-terminal region of TdT (del 2; 150–509) containing the Pol X domain did bind to it. del 3 and del 4 did not bind to UbcH5a, suggesting that the entire C-terminal region containing the Pol X domain is required for TdT binding to UbcH5a.

Knowing that TdT (del 2; 150–509) bound directly to UbcH5a, we carried out *in vitro* ubiquitylation assays using it. As shown in Figure 2C, del 2 was ubiquitylated by UbcH5a, suggesting that the region between residues 150–509 in TdT is ubiquitylated by UbcH5a.

Two molecular mechanisms are possible for TdT poly-ubiquitylation. TdT is poly-ubiquitylated either at a single or at multiple lysine residues. To investigate, we used methylated-ubiquitin, which lacks free amino groups and cannot form a poly-ubiquitin chain. Thus, if TdT were poly-ubiquitylated by methylated-ubiquitin or lysine-less ubiquitin (K0 Ub), TdT would have multiple sites for ubiquitylation. As shown in Figure 2D (lanes 4 and 5), only mono-ubiquitylated TdT was detected, indicating that TdT is poly-ubiquitylated at a single lysine residue.

Polyubiquitin chains are usually synthesized by ubiquitylation of the preceding ubiquitin on K48. We asked whether K48 in ubiquitin is required for TdT poly-ubiquitylation using a mutant, K48R. As shown in Figure 2D (lanes 2 and 3), TdT was poly-ubiquitylated by both wild type Ub and mutant K48R in the absence of E3. Thus, TdT is poly-ubiquitylated *in vitro* via lysine residues other than K48.

We next attempted to identify which lysine residue in TdT is ubiquitylated, by constructing TdT mutants. As shown in Figure 2C, the region between amino acid residues 150–509, which contains twenty three lysine residues that are potential candidates for TdT ubiquitylation, is likely to contain the ubiquitylation site. Among the lysines in this region are K199, K261, and K265, which are evolutionarily conserved. To determine the ubiquitylated lysine residue, we constructed seventeen TdT mutants, in which a lysine residue is replaced by arginine (described as TdT KxxxR). However, to our surprise, all the mutants were ubiquitylated (Figure 2E), even in the presence of BPOZ-2/Cul3 complex (data not shown). These results show that although TdT contains a single ubiquitylation site, any lysine residue in the 150–509 aa region can be ubiquitylated.

### Binding Specificity Between E2 and TdT

**Figure 3:**
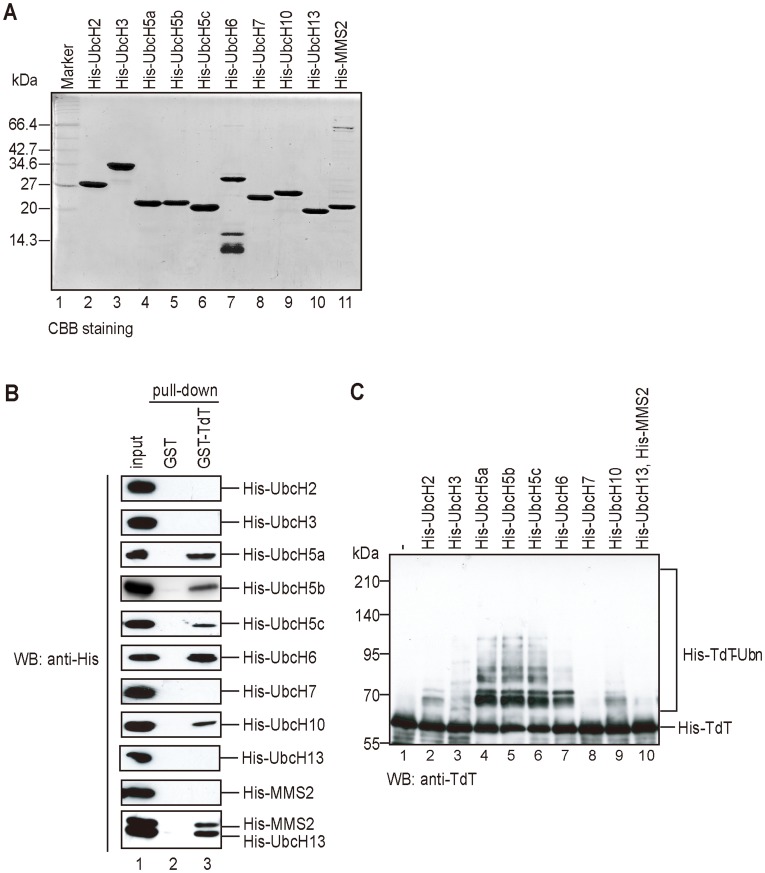
UbcH5a or UbcH6 directly binds to TdT and E3-independently ubiquitylates TdT *in vitro*. (A) Ten E2 enzymes were subjected to SDS-PAGE and stained by CBB. (B) Binding between TdT and E2 enzymes *in vitro*. Ten purified recombinant His-E2 enzymes were incubated with GST- (lane 2), GST-TdT (lane 3) bound Glutathione Sepharose 4B. Proteins bound to the beads were eluted with Laemmli buffer after boiling. The eluates were subjected to SDS-PAGE and detected by immunoblotting using an anti-His antibody. (C) E3-independent TdT ubiquitylation was carried out by 10 E2 enzymes. The substrate His-TdT was incubated with His-Ub, His-UBE1, and His-tagged E2 as indicated (lanes 2–10). After electrophoresis in a denaturing 7.5% polyacrylamide gel, ubiquitylated TdT was detected using an anti-TdT antibody.

To determine whether TdT binds to E2s other than UbcH5a, we chose UbcH5b (class I), UbcH5c (class II), and UbcH13, UbcH2 and UbcH3, UbcH6 and UbcH10 (class III). We also chose MMS2, which is an E2 variant that lacks a conserved, active cysteine [23] and forms a heteromeric complex with UbcH13 to produce K63-polyubiquitin chains [24]. These nine E2 cDNAs and UbcH5a cDNA were cloned, their gene products expressed as His-tagged proteins in *E. coli*, and purified (Figure 3A). The purified E2s were confirmed to form thiolesters with His-Ub (data not shown).

We then investigated the direct binding between TdT and E2s by GST pull-down assay using TdT as the bait. As shown in Figure 3B, the UbcH5 isoforms, UbcH6, UbcH10, and Ubc13/MMS2 bound to TdT, indicating that specific E2s can bind to TdT. We therefore expected that E2s that bind to TdT also ubiquitylate it. Therefore, we attempted to ubiquitylate TdT using the nine E2s, *in vitro*. As shown in Figure 3C, TdT was ubiquitylated by the UbcH5 isoforms and UbcH6, and at low levels by UbcH2 and UbcH10 (Figure 3C). These results are consistent with the binding specificity between the E2s and TdT.

### The SPA Motif in UbcH5a is Essential for Binding to TdT and TdT Ubiquitylation

**Figure 4:**
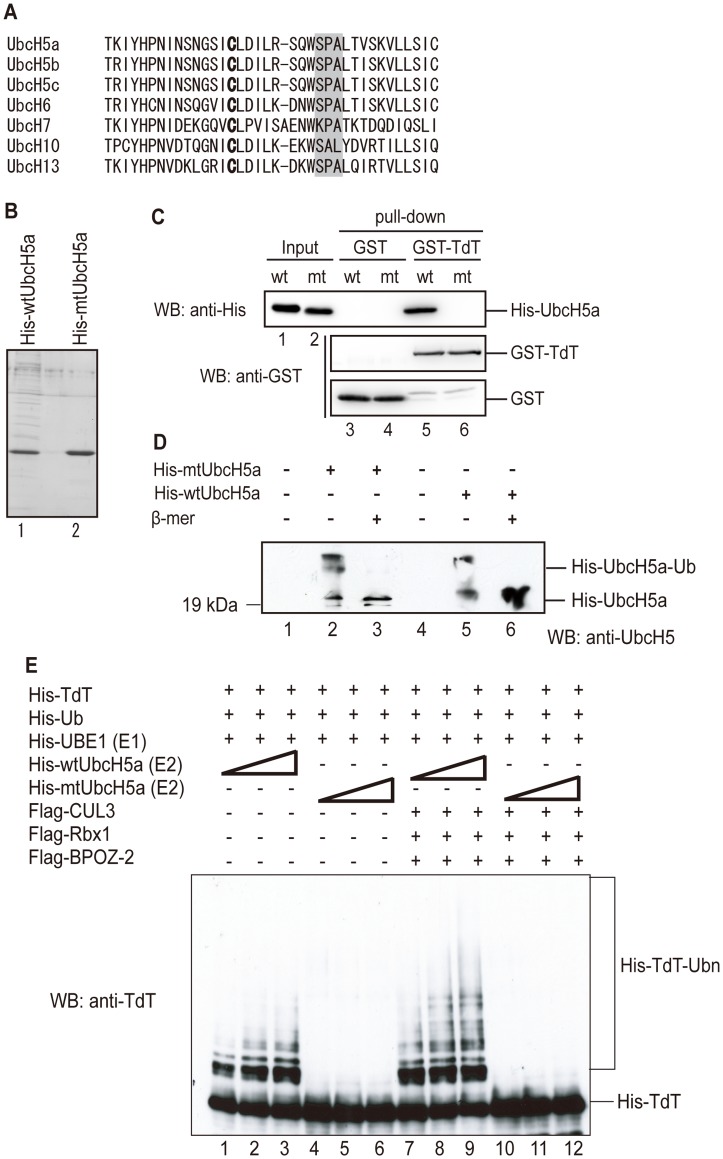
The SPA motif in UbcH5a is essential for binding to TdT and for TdT ubiquitylation. (A) Amino acid sequence alignment of E2s. The catalytic cysteine is indicated in boldface; the SPA motif is indicated by the gray background. UbcH5, UbcH6, and UbcH13, which bind to TdT, have a SPA motif in loop 7. (B) Mutation of the SPA motif in UbcH5a to AAA. The UbcH5a mutant (mtUbcH5a) was expressed in *E. coli* as a His-tagged protein and purified. His-mtUbcH5a (lane 1) and His-wtUbcH5a (lane 2) were subjected to SDS-PAGE and stained with Coomassie Brilliant Blue (CBB). (C) Reduction of UbcH5a’s binding ability by the mutated SPA motif. His-wtUbcH5a and His-mtUbcH5a were incubated with Glutathione Sepharose 4B-bound GST (lanes 3 and 4, respectively) or GST-TdT (lanes 5 and 6, respectively). Proteins bound to the beads were eluted by boiling with Laemmli buffer and immunoblotted with an anti-His or anti-GST antibody. (D) Formation of thioester adducts of wtUbcH5a and mtUbcH5a expressed in *E. coli*. The thioester reaction mixture contained Ub, UBE1, and UbcH5a as indicated. The same amount of each UbcH5a was used for CBB staining. After 5 min at RT, reactions were stopped by adding Laemmli buffer with (lanes 3 and 6) or without (lanes 1, 2, 4, and 5) β-mercaptoethanol, and proteins were separated by SDS-PAGE. UbcH5a thioester adducts were detected by immunoblotting using an anti-UbcH5 antibody. (E) TdT is not ubiquitylated by mtUbcH5a. E3-independent (lanes 1 to 6) or Cul3-dependent TdT ubiquitylation by the BPOZ-2/Cul3/Rbx1 complex (lanes 7 to 12) was carried out. TdT was incubated in a ubiquitylation reaction mixture containing E1, ubiquitin, and wtUbcH5a or mtUbcH5a. His-TdT ubiquitylation was detected by immunoblotting with an anti-TdT antibody.

Because of the binding specificity between TdT and E2s, we expected that E2s possess a unique determinant for TdT binding. The E3 ligase CHIP binds to three types of E2, UbcH5, Ubc13-Uev1a, and Ube2e2, through an SPA motif in loop L7 of the cognate E2 [25]. The SPA motif makes a hydrogen bond with the carbonyl group of CHIP-P269 and van der Waals contacts with CHIP-H260, V264, and V270. We searched for SPA motifs in loop L7 in E2s, and found that the UbcH5 isoforms, UbcH6, and UbcH13 contained a typical SPA motif (Figure 4A).

To determine whether E2 binds to TdT through the SPA motif, we constructed a UbcH5a mutant (mtUbcH5a) containing two amino acid substitutions in the SPA motif (S94A, P95A), expressed the mutants as His-tagged proteins in *E. coli,* and purified them (Figure 4B). We then examined direct binding between TdT and mtUbcH5a by GST pull-down assay, using TdT as the bait. As shown in Figure 4C, mtUbcH5a did not bind to TdT, indicating that E2 binds to TdT through the SPA motif in E2.

We next examined whether mtUbcH5a could ubiquitylate TdT *in vitro.* We first confirmed that mtUbcH5a was able to form a thiolester bond with His-Ub (Figure 4D). We then incubated TdT with increasing concentrations of wild-type UbcH5a (wtUbcH5a) or mtUbcH5a. We found increasing amounts of ubiquitylated TdT with increasing amounts of wtUbcH5a, but not with increasing mtUbcH5a (Figure 4E; lanes 1 to 6). Moreover, TdT ubiquitylation was promoted by BPOZ-2/Cul3 complexed with wtUbcH5a, whereas TdT was not ubiquitylated by mtUbcH5a, even in the presence of the BPOZ-2/Cul3 complex (Figure 4E; lanes 7 to 12). These results show that the SPA motif in UbcH5a is necessary for TdT ubiquitylation. Since UbcH13, which contains a SPA motif, binds to TdT (Fig. 3B) but does not ubiquitylate it (Fig. 3C), the existence of a SPA motif in the E2 is not sufficient for TdT ubiquitylation.

### TdT Binds to UbcH5 and UbcH6 in 293 T Cells

**Figure 5:**
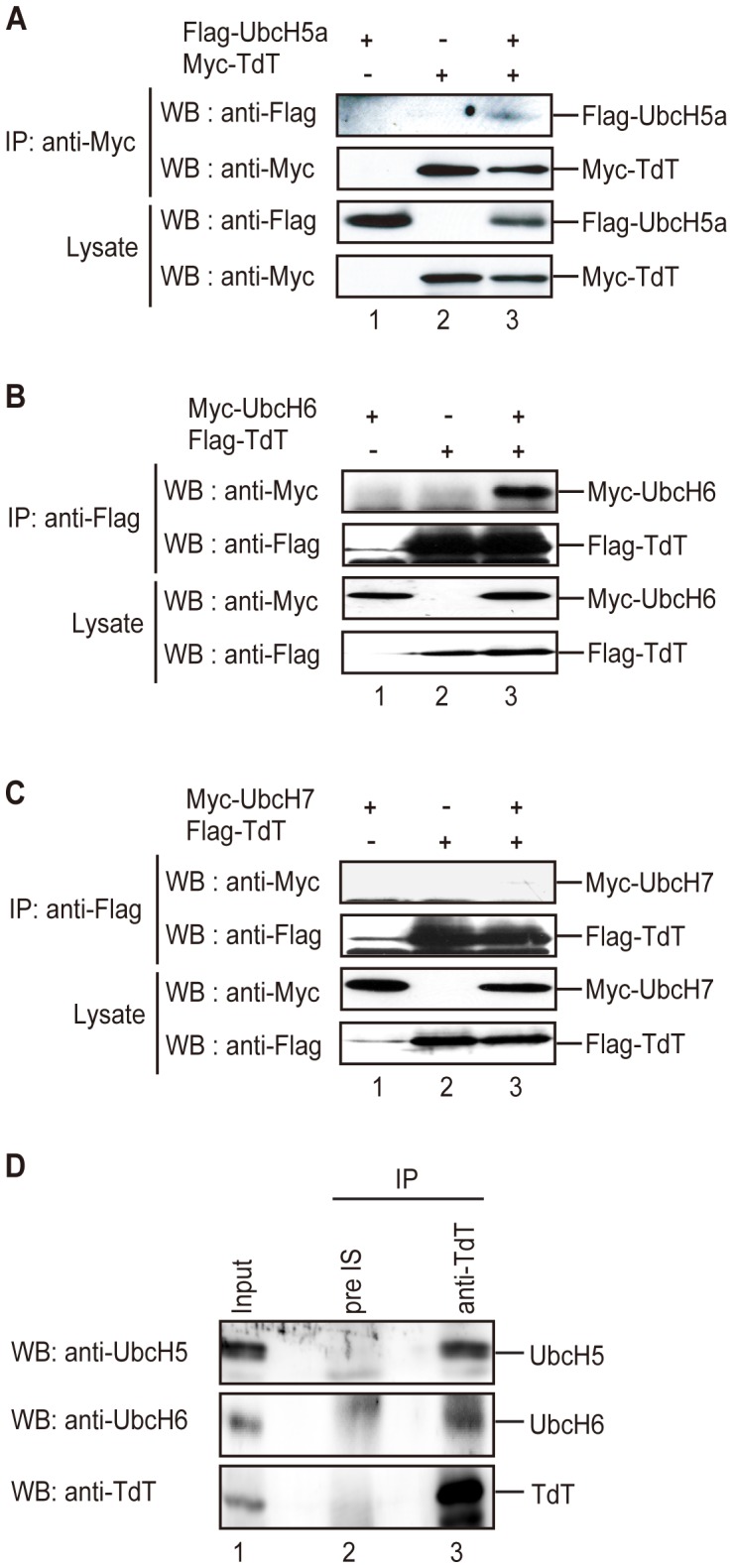
UbcH5a or UbcH6 binds to TdT *in vivo*. (A) Immunoprecipitation using Myc-TdT as the bait. 293 T cells were co-transfected with expression vectors encoding Flag-UbcH5a (lanes 1 and 3) and Myc-TdT (lanes 2 and 3). Immunoprecipitation was carried out using an anti-Myc antibody. Immunoprecipitants were subjected to SDS-PAGE and following immunoblotting using an anti-Flag or anti-Myc antibody. (B) Immunoprecipitation using Flag-TdT as the bait. 293 T cells were co-transfected with expression vectors encoding Myc-UbcH6 (lanes 1 and 3) and Flag-TdT (lanes 2 and 3), and immunoprecipitation was carried out using an anti-Flag antibody. Immunoprecipitants were separated by SDS-PAGE and immunoblotted with an anti-Flag or anti-Myc antibody. (C) Immunoprecipitation using Flag-TdT as the bait. 293 T cells were co-transfected with expression vectors encoding Myc-UbcH7 (lanes 1 and 3) and Flag-TdT (lanes 2 and 3). Immunoprecipitation using an anti-Flag antibody. Immunoprecipitants were separated by SDS-PAGE and immunoblotted with an anti-Flag or anti-Myc antibody. (D) Immunoprecipitation of endogenous TdT in Jurkat cell lysates, using an anti-TdT antibody (lane 2) or rabbit pre-immune serum (pre IS) (lane 3). Immunoprecipitants were separated by SDS-PAGE and immunoblotted with an anti-UbcH5, anti-UbcH6 or anti-TdT antibody. Each immunoprecipitation used 1 mg whole cell lysate.

To confirm the association between TdT and E2 in cells, we performed immunoprecipitation assays using 293 T cells expressing Flag-tagged UbcH5a and Myc-tagged TdT, Myc-tagged UbcH6 and Flag-tagged TdT, or Myc-tagged UbcH7 and Flag-tagged TdT. As shown in Figure 5A, UbcH5a and UbcH6 bound to TdT (Figure 5B), but UbcH7 did not (Figure 5C). These results were consistent with the *in vitro* binding specificities of TdT for particular E2s.

We further determined whether endogenous TdT associates with UbcH5a or UbcH6. TdT expressed by Jurkat cells was precipitated using an anti-TdT antibody, and the presence of an associated UbcH5a and UbcH6 was detected by immunoblotting with anti-UbcH5 and anti-UbcH6 antibodies. As shown in Figure 5D, the UbcH5 isoform and UbcH6 were detected in the TdT precipitants in asynchronously proliferating MOLT4 cells. Since the amino acid sequences of the UbcH5 isoforms exhibit high homology (UbcH5a is 89 and 88% identical to UbcH5b and UbcH5c, respectively; [26], [27]), an anti-UbcH5 antibody used in this study could not distinguish among the UbcH5 isoforms.

### UbcH5a and UbcH6 Promote TdT Ubiquitylation

**Figure 6:**
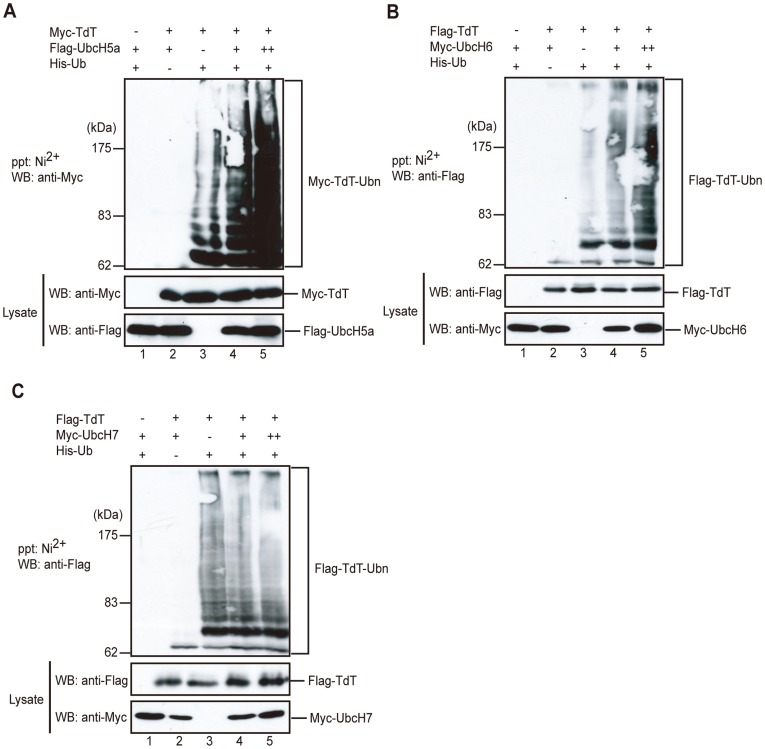
UbcH5a and UbcH6 promote TdT ubiquitylation. (A) UbcH5a enhances TdT ubiquitylation in 293 T cells. 293 T cells were transfected with plasmids encoding His-Ub (lanes 1, 3 to 5), Myc-TdT (lanes 2 to 5), and/or Flag-UbcH5a (lanes 1, 2, 4 and 5) in the indicated combinations. After a 24 h incubation, the cells were treated with 10 µM MG132 for another 12 h, lysed under denaturing conditions, and the ubiquitylated proteins were affinity-purified and separated by SDS-PAGE. Ubiquitylated Myc-TdT was detected by immunoblotting with an anti-Myc antibody. Myc-TdT and Flag-UbcH5a in the lysate were detected using an anti-Myc or anti-Flag antibody. (B) UbcH6 enhances TdT ubiquitylation in 293 T cells. 293 T cells were transfected with plasmids encoding His-Ub (lanes 1, 3 to 5), Flag-TdT (lanes 2 to 5), and/or Myc-UbcH6 (lanes 1, 2, 4 and 5) in the indicated combinations. After incubation for 24 h, the cells were treated with 10 µM MG132 for another 12 h. The cells were lysed under denaturing conditions, and the ubiquitylated proteins were affinity-purified and separated by SDS-PAGE. Ubiquitylated TdT was detected by immunoblotting using an anti-Flag antibody. Flag-TdT and Myc-UbcH6 in the lysate were detected using an anti-Flag or anti-Myc antibody. (C) UbcH7 does not enhance TdT ubiquitylation in 293 T cells. 293 T cells were transfected with plasmids encoding His-Ub (lanes 1, 3 to 5), Flag-TdT (lanes 2 to 5), and/or Myc-UbcH7 (lanes 1, 2, 4 and 5) in the indicated combinations. After a 24 h incubation, the cells were treated with 10 µM MG132 for another 12 h, lysed under denaturing conditions, and the ubiquitylated proteins were affinity-purified and separated by SDS-PAGE. Ubiquitylated TdT was detected by immunoblotting using an anti-Flag antibody. Flag-TdT and Myc-UbcH7 in the lysate were detected using an anti-Flag or anti-Myc antibody.

We next determined whether TdT is a target of UbcH5a-mediated ubiquitylation. Myc-tagged TdT and/or Flag-tagged UbcH5a, together with His-tagged ubiquitin, were expressed in 293 T cells and subjected to immunoblot analysis using an anti-Myc antibody. As shown in Figure 6A, TdT was ubiquitylated in 293 T cells when co-expressed with ubiquitin, and Ubch5a markedly enhanced TdT ubiquitylation. We further determined that UbcH6 also promoted TdT ubiquitylation *in vitro*, while UbcH7 did not (Figure 6B and 6C). Thus, UbcH5a and UbcH6 promote TdT ubiquitylation in 293 T cells.

### Knockdown of Endogenous UbcH5 Isoforms or UbcH6 Reduces TdT Ubiquitylation

**Figure 7:**
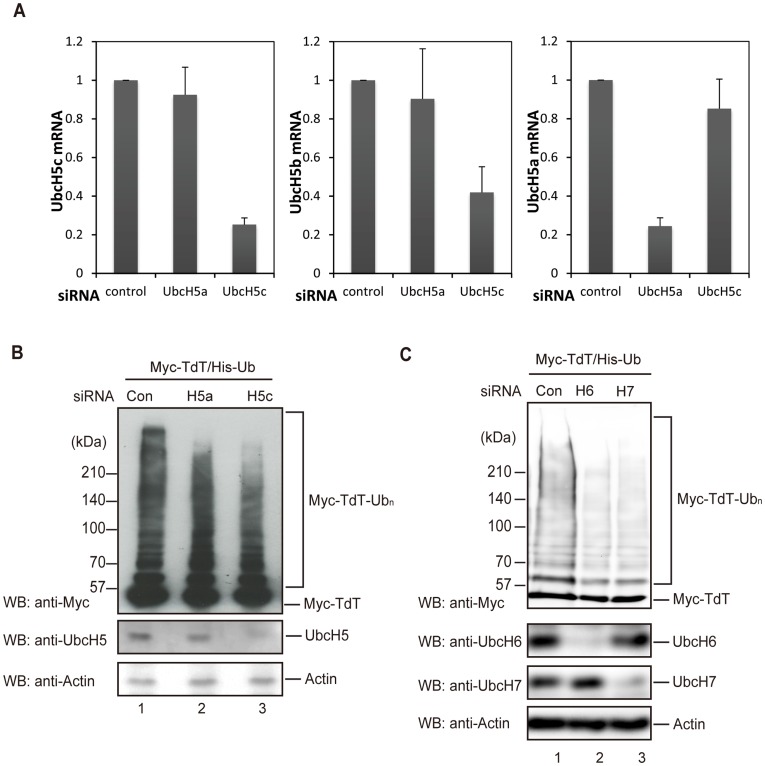
Knockdown of UbcH5, UbcH6, or UbcH7 mRNA reduces TdT ubiquitylation. (A and B) 293 T cells were transfected with a control siRNA or siRNAs targeting UbcH5a or UbcH5c using the X-tremeGENE Transfection reagent 24 h prior to the plasmid transfection. The cells were then transfected with Myc-TdT and His-Ub using the GeneJuice Transfection Reagent. After a 24 h incubation, the cells were treated with 10 µM MG132 for another 12 h. mRNA levels of UbcH5 isoforms were measured by real-time PCR, normalized to GAPDH, and are expressed as their ratio to cells transfected with a control siRNA (A). Ubiquitylated Myc-TdT and UbcH5 in the lysate were detected by immunoblotting with an anti-Myc, anti-UbcH5, or anti-actin antibody (B). (C) 293 T cells were transfected with a control siRNA or siRNAs targeting UbcH6 or UbcH7 using the X-tremeGENE Transfection reagent 24 h prior to the plasmid transfection. The cells were then transfected with Myc-TdT and His-Ub using the GeneJuice Transfection Reagent. After incubation for 24 h, the cells were treated with 10 µM MG132 for another 12 h. Ubiquitylated Myc-TdT, UbcH6, and UbcH7 in the lysate were detected by immunoblotting with an anti-Myc, anti-UbcH6, anti-UbcH7, or anti-actin antibody.

To further confirm the TdT ubiquitylation by UbcH5 *in vivo*, we reduced the endogenous UbcH5 in 293 T cells with specific UbcH5 siRNAs. We targeted UbcH5a, UbcH5b or UbcH5c and quantified TdT ubiquitylation in 293 T cells expressing Myc-TdT and His-Ub. Due to the high degree of homology and the functional redundancy between UbcH5b and UbcH5c, we generated single siRNA duplexes that could reduce both UbcH5b and UbcH5c (UbcH5b/c). As shown in Figure 7A, the UbcH5a, UbcH5b, and UbcH5c mRNAs were reduced to 22%, 40%, or 22% of the original amount, respectively. As shown in Figure 7B, total UbcH5 was reduced to 50% by knock-down of Ubc5b/c mRNA (lane 3), whereas only 5% of UbcH5 was reduced by knock-down of UbcH5a mRNA (lane 2), suggesting that the amount of UbcH5b/c is higher than that of UbcH5a in 293 T cells.

Although the reduction of UbcH5a by its specific siRNA resulted in a slight reduction of TdT ubiquitylation in 293 T cells (lane 2), the knockdown of UbcH5c substantially reduced TdT ubiquitylation (lane 3). Thus, UbcH5b/c appear to preferentially mediate TdT ubiquitylation in 293 T cells, compared to UbcH5a. From the ubiquitylation assay and binding analysis, we expected UbcH6 to be a potent E2 for BPOZ-2/Cul3 complex-independent TdT ubiquitylation. We therefore investigated the effects of the targeted knockdown of UbcH6 or UbcH7 mRNAs on TdT ubiquitylation, by specific siRNAs for UbcH6 or UbcH7 and quantified TdT ubiquitylation in total cell lysates of 293 T cells expressing Myc-TdT and His-Ub by immunoblotting. As shown in Figure 7C, the knockdown of UbcH6 mRNA markedly reduced the TdT ubiquitylation compared to the effect of a control siRNA, indicating that UbcH6 mediates TdT ubiquitylation in 293 T cells. Unexpectedly, knockdown of UbcH7 mRNA also reduced TdT ubiquitylation, even though UbcH7 does not bind directly to TdT *in vitro* (Figure 5) or *in vivo* (Figure 6). UbcH7 may form a complex with E3 or other factor(s) *in vivo*, thus acquiring ubiquitylation activity for TdT. These findings suggest that TdT is ubiquitylated by multiple pathways related to UbcH5, UbcH6, and UbcH7, respectively. To examine the possibility, we performed double and triple siRNA knock down of the above players and quantified TdT ubiquitylation in 293 T cells expressing Myc-TdT and His-Ub. Expectedly, TdT ubiquitylation was dramatically decreased when UbcH5, UbcH6 and UbcH7 were depleted by triple siRNA knock down (Fig. S1), strongly suggesting that TdT is ubiquitylated by multiple pathways including UbcH5, UbcH6, and UbcH7.

### Ubiquitylation of TdT Inhibits its Nucleotidyltransferase Activity

**Figure 8:**
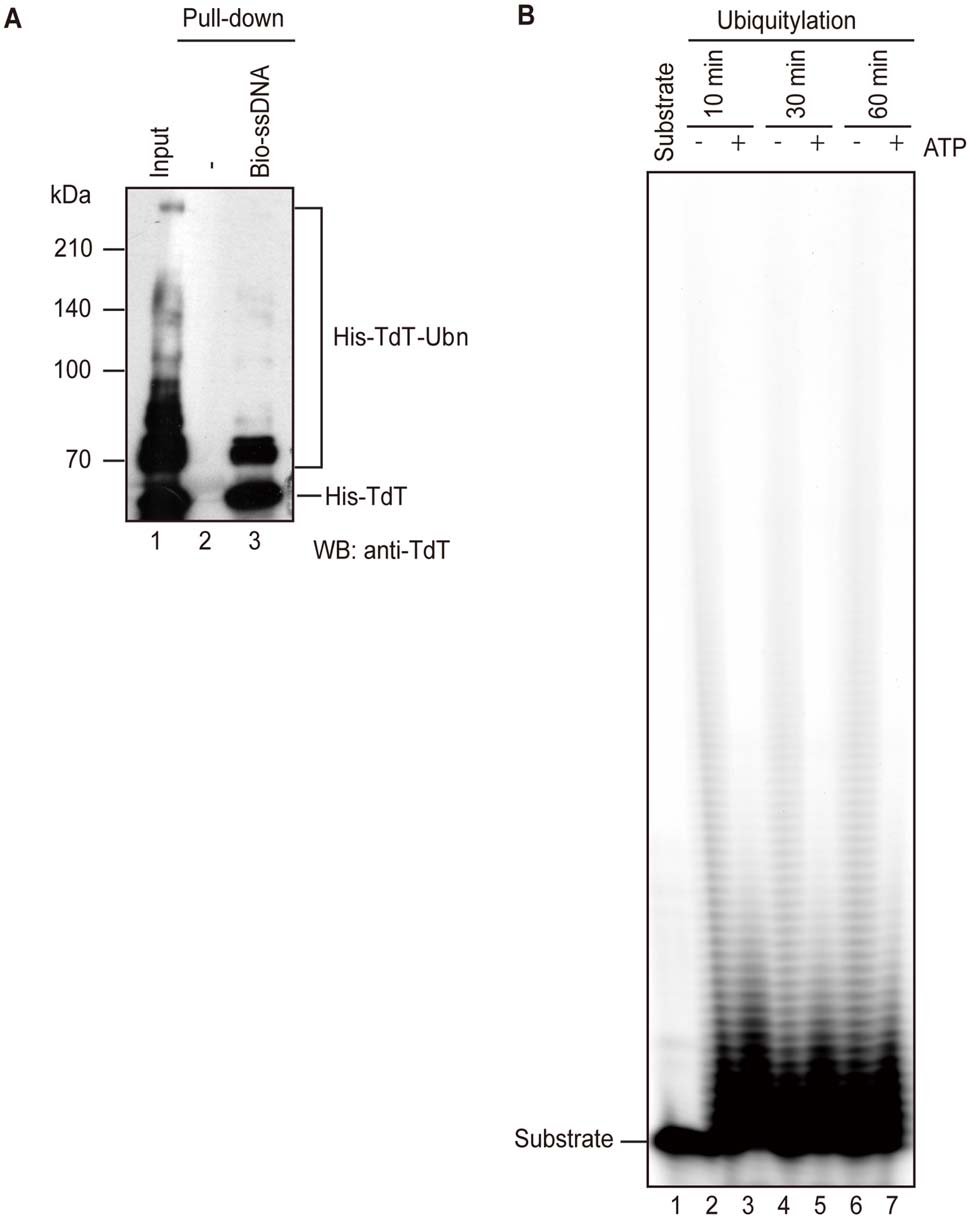
TdT ubiquitylation inhibits its nucleotidyltransferase activity. (A) Ubiquitylation of TdT does not effect its DNA-binding property. His-TdT (180 ng) was incubated in the reaction *in vitro* ubiquitylation mixture with (lane 3) or without (lane 2) biotinylated ssDNA coupled to streptavidin–agarose. The proteins that bound to DNA were subjected to immunoblot analysis using an anti-TdT antibody. (B) Primer extension assay. GST-TdT bound Glutathione Sepharose 4B was added to the ubiquitylation reaction mixture and then incubated with (lanes 2, 4, and 6) or without (lanes 3, 5, and 7) ATP, for the indicated times. After washing, primer extension was performed in reaction mixture containing the Cy5-labeled 20-mer oligo-dT. After 8% SDS page, the products were visualized on a Typhoon 9200 Gel Imager (GE Healthcare).

Ubiquitylation can have many effects, including suppressing the ability of the target protein to bind DNA [28]. We examined the DNA binding and nucleotidyltransferase activities of ubiquitylated TdT. To assess TdT’s DNA binding activity, a synthetic biotinylated-oligonucleotide was immobilized on streptavidin-agarose and the ubiquitylation reaction was carried out in the presence of ssDNA-coupled beads. The proteins that bound to the ssDNA were subjected to immunoblotting to detect TdT. As shown in Figure 8A, both TdT and ubiquitylated TdT bound to the ssDNA-coupled streptavidin-agarose (lane 3), but BPOZ-2 and Cul3 did not bind to it (data not shown). When using the purified sample containing highly ubiquitylated TdT from 293 T cells (Figure S2A), ubiquitylated TdT also bound to the ssDNA (Figure S2B). Thus, ubiquitylation did not abolish TdT’s DNA-binding ability.

We next determined whether ubiquitylated TdT maintains its nucleotidyltransferase activity by using a primer extension assay and ubiquitylated GST-TdT. We found that when TdT was incubated in the ubiquitylation mixture without ATP, ssDNA was effectively elongated by TdT (Figure 8B, lanes 2, 4, and 6). However, the amount of primer extension was reduced when TdT was incubated in the ubiquitylation mixture with ATP (lanes 3, 5 and 7). We also confirmed that TdT ubiquitylation increased with the duration of the ubiquitylation reaction (data not shown), consistent with the ubiquitylation of TdT inhibiting its nucleotidyltransferase activity. Moreover?we measured the activity using purified sample containing ubiquitylated TdT from 293 T cells (Figure S2A). TdT activity was not detected when using the purified sample containing highly ubiquitylated TdT (Figure S2C). The nucleotidyltransferase activity of unmodified TdT might be inhibited by ubiquitylated TdT in a competitive manner, because ubiquitylated TdT retains DNA binding activity.

## Discussion

Here we showed that TdT is ubiquitylated without E3 by directly binding to E2, and that ubiquitylated TdT’s nucleotidyltransferase activity is inhibited by ubiquitylation, *in vitro*. Down regulation of UbcH5 or UbcH6 in 293 T cells markedly reduced TdT ubiquitylation, indicating that E2-mediated ubiquitylation is critical for TdT ubiquitylation *in vivo*. Two proteins have been reported to be poly-ubiquitylated by E2 without E3. Ataxin-1 and Zipper-interacting protein kinase (ZIPK) are ubiquitylated by UbcH6 [29] and UbcH5c [30], respectively. TdT is the third protein and the first DNA polymerase found to be ubiquitylated only by E2. Six proteins, STAM2, Eps15, pol ι, pol κ, HDAC6, and Sts1 are mono-ubiquitylated by E2 without E3 [31]. Since the BPOZ-2/Cul3 complex enhances TdT ubiquitylation [4], the complex may function as an E3 during the second ubiquitylation step after the first ubiquitylation step by an E2. E3 may enhance ubiquitylation after cells receive a signal for a second ubiquitylation, since the Cul3-based E3 complex commonly recruits substrates after an inducible post-translational modification, such as neddylation.

Here we showed that ubiquitylation of TdT functions as a signal for TdT degradation and suppression of TdT activity. Ubiquitylation of proteins regulates protein-protein and protein-DNA interactions for purposes other than protein degradation. Vertebrate Y-family DNA polymerases preferentially interact with the mono-ubiquitylated form of PCNA [32], [33]. The ubiquitylation of DDB2 (damaged DNA binding protein 2) alters the DNA binding property of UV-DDB *in vitro* [28]. Our finding is the first to suggest that the ubiquitylation of protein affects enzymatic activity. Toxic cellular enzymes, such as TdT, may be inactivated first by ubiquitylation by an E2 and then further ubiquitylated by an E3 to accelerate protein degradation.

UbcH5 or Ubc13 bind to the C-terminus of Hsp70 interacting protein (CHIP) through an SPA motif [25]. We found that the SPA motif in loop L7 of UbcH5a is required to bind to and ubiquitylate TdT. The SPA motif mutant mtUbcH5a neither bound to nor ubiquitylated TdT *in vitro*. Since UbcH5b, UbcH5c, UbcH6, and UbcH13-MMS2 also have SPA motifs and bind to TdT, they may also bind TdT through their SPA motifs. In addition, the class III E2 enzymes, Ube2e2 and Ube2e3, which are UbcH6 homologues and have a SPA motif, should be able to bind to TdT. Since UbcH13-MMS2 binds to TdT but does not ubiquitylate it, the SPA motif might be necessary but not sufficient for TdT ubiquitylation. Surprisingly, UbcH10 binds to TdT and ubiquitylates it *in vitro*, although UbcH10 contains alanine and leucine at the positions corresponding to the proline and alanine in the SPA motif, respectively. Of them, proline is essential for the SPA motif function. Residues other than proline in the SPA motif probably provide the TdT binding and ubiquitylation activities.

Proteins are usually ubiquitylated by E2 and E3. However, some E3s are auto-ubiquitylated [34]. Furthermore, since E2 binds to TdT through the SPA motif, and since TdT is ubiquitylated by E2 without E3, TdT itself may function as an E3. TdT consists of two functionally different regions, the N-terminal region, containing the BRCT domain, and the C-terminal pol β-like region. We found that the entire pol β-like region is required for the binding and ubiquitylation of TdT by UbcH5a. Thus, the pol β-like region is probably functionally similar to the HECT (homologous to E6-AP carboxyl terminus), RING (really interesting new protein) finger, PHD (plant homeodomain)-like, or U-box domains, which are E2-binding domains in E3s [35], [36], [37]. However, at present we have no evidence that TdT has substrates for ubiquitylation.

TdT’s major ubiquitylation site is in the pol β-like region, which contains many lysine residues (28 residues in humans; 30 residues in mice). From comparisons of the amino acid sequences of the pol β-like regions of human, mouse, cow, opossum, chicken, African clawed frog, and zebrafish TdT, we selected 23 highly conserved lysine residues and mutated them. Since all of the TdT mutants were ubiquitylated, no lysine-specific targeting appears necessary for ubiquitylation in the pol β-like region. Poly-ubiquitylation may depend on an initial mono-ubiquitylation on a single lysine residue in the pol β-like region, on which a poly-ubiquitin chain may be formed. It may be physically difficult for poly-ubiquitin chains to be formed simultaneously on multiple lysine residues in the pol β-like region, because of the proximity of the lysine residues to one another. As shown in Figure 2D (lane 5), a faint band of TdT ubiquitylation at a second lysine residue was detected. This secondary ubiquitylation may be necessary for reliable and rapid TdT ubiquitylation to regulate both TdT activity and concentration in a cell. Even if a mutation is induced at one lysine residue, other lysines assure TdT ubiquitylation.

Like TdT, Nrf2, which is a substrate of the Keap1/Cul3 E3 complex, contains multiple (seven) lysine residues targeted for ubiquitylation within a single domain, the Neh2 domain [38], and which lysine residue is ubiquitylated is not specifically determined in advance. If ubiquitinylation were restricted to a unique lysine, its mutation would crucially affect cell survival. Thus, cells are protected from the toxic TdT activity by prompt TdT degradation following polyubiquitylation at any of the multiple ubiquitylation sites.

We have analyzed the nature of TdT ubiquitylation by establishing an *in vitro* ubiquitylation system. We are also attempting to determine whether TdT is ubiquitylated by E2 without E3 in vivo. Since TdT synthesizes the N region of Ig and T-cell receptor genes, we would like to clarify the biological significance of TdT ubiquitylation during V(D)J recombination.

## Materials and Methods

### Plasmids

TdT cDNA was isolated from a bovine thymus cDNA library [39]. Expression plasmids of human genes encoding BPOZ-2, Rbx-1, UbcH3, UbcH5a, UbcH5b, UbcH5c, UbcH6, UbcH7, UbcH10, UbcH13 and MMS2 were constructed after amplifying their cDNAs in the human thymus cDNA library by PCR. UbcH2 gene was amplified from a human liver cDNA library. UBE1 was isolated from cDNA library of HeLa cells (ATCC). Cul3 cDNA is a kind gift from Dr. T. Nagase (Kazusa DNA Research Institute, Chiba, Japan). The lysine-less ubiquitin (K0 Ub) cDNA is a kind gift from Dr. S. Nakada (Keio Univ., Tokyo, Japan). The His-tagged Ub expression vector, pMT107, is a kind gift from Dr. D. Bohmann [40]. Site-directed mutagenesis was performed to obtain various TdT mutants and a UbcH5a mutant was acquired using PrimeSTAR Max (TaKaRa) according to the manufacturer’s instructions.

### Antibodies and Chemical Reagents

The rabbit polyclonal antibody (pAb) against TdT was raised using purified calf TdT. Mouse monoclonal antibodies (mAbs) against the Flag epitope tag (M2), actin (AC40), and ubiquitin (6C1) were obtained from Sigma; mouse mAbs against mono- and poly-ubiquitylated conjugates (FK2) from BIOMOL; mouse mAbs against the Myc epitope tag (4A6) from Upstate; rabbit pAb against the GST epitope tag from Affinity BioReagents; rabbit pAb against UbcH5, UbcH6, and UbcH7 from Boston Biochem; mouse mAb against penta-His from QIAGEN; and secondary anti-mouse and anti-rabbit IgG antibodies conjugated with HRP from New England Biolabs. Ubiquitin aldehyde (BIOMOL), methylated ubiquitin (Boston Biochem), and MG132 (Sigma) were purchased.

### Cell Culture and Transient Transfection

293 T (*human* embryonic kidney) and Jurkat cells (T Lymphoma) were obtained from the ATCC. 293 T cells were cultured in Dulbecco’s modified Eagle medium (Gibco-BRL) supplemented with 10% fetal bovine serum and 100 µg/ml kanamycin. Jurkat cells were cultured in RPMI 1640 supplemented with 10% fetal bovine serum and 100 µg/ml kanamycin. Transfections were performed following the manufacturer’s instructions: the Gene Juice (Merck) transfection reagent was used for plasmids; X-tremeGENE siRNA Transfection Reagent (Roche) was used for RNA.

### Proteins

Recombinant wild type (wt) and mutant His-TdT, GST-TdT, His-BPOZ-2, and His-UBE1 proteins were expressed in *E. coli* strain BL21(DE3)pLysS Rosetta2 (Novagen). The transformed cells were grown at 37°C until 0.6 OD600, and expression was induced by 100 µM isopropyl-1-thio-β-D-galactopyranoside for an additional 20 h at 22°C. Recombinant His-Ub, His-Ub K48R, His-K0 Ub, His-UbcH2, His-UbcH3, His-UbcH5a WT, and the mutant constructs GST-Ubc5a, His-UbcH5b, His-UbcH5c, His-UbcH6, His-UbcH7, His-UbcH10, His-UbcH13, His-MMS2 and GST-RNase III, were expressed in *E. coli* strain BL21(DE3). The transformed cells were grown at 37°C until 0.6 OD600 and then expression was induced by 400 µM isopropyl-1-thio-β-D-galactopyranoside for an additional 3 h at 37°C. Cells were harvested by centrifugation at 4,000 g for 5 min. The cell pellets were resuspended in the lysis buffer A (50 mM Tris-HCl pH 7.4, 150 mM NaCl, 1% TritonX-100, 10% glycerol, 1 mM DTT, 1 mM PMSF, 1 mg/ml pepstatin A, 1 mg/ml leupeptin, and 5 mM benzamidine) and disrupted by sonication. Insoluble material was pelleted by centrifugation for 20 min at 22,000 g.

For His-tagged E2 and His-Ub, the cell pellets were resuspended in lysis buffer B (50 mM sodium phosphate pH 8.0, 300 mM NaCl, 0.2% TritonX-100, 10% glycerol, 5 mM β-mercaptoethanol, 1 mM PMSF, 1 mg/ml pepstatin A, 1 mg/ml leupeptin, and 5 mM benzamidine). Cell lysates containing His-Ub, His-Ub K48R, or His-K0 Ub were boiled for 10 min, followed by centrifugation to remove insoluble material. His-tagged and were purified using Ni Sepharose 6 Fast Flow (GE Healthcare) and GST-fused proteins were purified using Glutathione Sepharose 4B (GE Healthcare), according to the manufacturer’s instructions.

Flag-tagged proteins were transiently expressed in 293 T cells. 293 T cells were transfected with plasmids encoding Flag-CUL3, Flag-Rbx1, Flag-BPOZ-2, or Flag-TdT by HilyMax (Dojindo) transfection reagent. Thirty-six hours after transfection, cells were lysed with lysis buffer C (50 mM Tris-HCl pH 7.5, 150 mM NaCl, 10% glycerol, 1% Nonidet P-40, 1 mM EDTA, 1 mM DTT, 1 mM PMSF, 5 µg/ml leupeptin, 10 µg/ml aprotinin, 1 mM benzamidine, and 2 µg/ml pepstatin A). Flag-tagged proteins were purified with ANTI-FLAG M2 affinity gel (Sigma) and eluted with 0.1 mg/ml Flag-peptide. Ubiquitylated Flag-TdT was purified from 293 T cells, which were co-transfected with plasmids encoding Flag-TdT and His-Ub. Thirty-six hours after the transfection, the cells were treated with MG132 (final concentration, 10 µM) for 7 h to abrogate proteasome activity. Cells were lysed with lysis buffer C containing MG132. The soluble fraction was incubated with Ni Sepharose 6 Fast Flow for 2 min to concentrate the poly-ubiquitylated proteins. Poly-ubiquitylated proteins containing poly-ubiquitylated Flag-TdT were eluted from the beads with 500 mM imidazole. Oligo-ubiquitylated Flag-TdT in the unbound fraction and poly-ubiquitylated Flag-TdT in the eluate was purified with ANTI-FLAG M2 affinity gel (Sigma).

### GST Pull-down Assay, Immunoprecipitation, and Immunoblotting

GST-fused proteins were coupled to glutathione Sepharose 4B beads (GE Healthcare) in binding buffer A (50 mM Tris-HCl (pH 7.4), 150 mM NaCl, 2.5 mM MgCl_2_, 1% TritonX-100, 10% glycerol, 1 mM DTT) by incubation for 1 h at 4°C. The beads were washed 3 times with binding buffer A and incubated with His-tagged proteins. After an additional incubation for 1 h, the beads were washed 5 times with wash buffer A (50 mM Tris-HCl pH 7.4, 150 mM NaCl, 2.5 mM MgCl_2_, 0.2% TritonX-100, 10% glycerol, 1 mM DTT), and bound proteins were analyzed by SDS-PAGE followed by immunoblotting using an anti-His or anti-GST antibody.

For immunoprecipitation, cellular proteins from 293 T cells, Jurkat cells, or the bovine thymocytes were extracted using lysis buffer D (50 mM Tris-HCl pH 7.5, 150 mM NaCl, 2.5 mM MgCl_2_, 10% glycerol, 0.5% Nonidet P-40, 1 mM DTT, 1 mM PMSF, 5 µg/ml leupeptin, 10 µg/ml aprotinin, 1 mM benzamidine, and 2 µg/ml pepstatin A). The cell lysates were incubated with the appropriate antibodies for 2 h at 4°C and then with 20 µl of a 50% slurry of protein A-Sepharose (GE Healthcare) or protein G-Sepharose (GE Healthcare) for another 1 h. After 5 washes with 1 ml of wash buffer Β (50 mM Tris-HCl (pH 7.5), 150 mM NaCl, 2.5 mM MgCl_2_, 10% glycerol, and 0.01% Nonidet P-40), bound proteins were eluted by boiling and subjected to SDS-PAGE and immunoblotting.

### *In vivo* Ubiquitylation Assay

293 T cells were transfected with several combinations of plasmids together with the His-Ub vector as described in Figure legends. Twenty-four hours after the transfection, the cells were treated with DMSO or MG132 (final concentration, 10 µM) for 12 h to abrogate proteasome activity. Whole cell extracts were prepared in lysis buffer U (20 mM Tris-HCl (pH 7.4), 500 mM NaCl, 0.02% Nonidet P-40, 8 M urea, and 5 mM imidazole) and incubated overnight on a HIS-Select Nickel Affinity Gel (Sigma). After four washes with lysis buffer U, the beads were boiled in the sample buffer and the eluate was subjected to immunoblotting using an anti-Myc or an anti-Flag antibody.

### *In vitro* Ubiquitylation Assay

Ubiquitylation was performed in a 10 µl reaction buffer containing 50 mM Tris pH 7.4, 5 mM MgCl_2_, 2 mM NaF, energy regeneration system (4 mM ATP, 3 U/µl creatine kinase, 10 mM creatine phosphate), Ub aldehyde (200 ng), 1 mM DTT, and 20 µM MG132. Where necessary, His-UBE1 (100 ng), His-Ub or Me-Ub (7.2 µg), His-tagged E2 (200 ng), His-TdT WT or mutant (60 ng), or Flag-Cul3/Flag-BPOZ-2/Flag-Rbx1 was included. Reactions were incubated at 37°C for 1 h, terminated by Laemmli buffer, and separated on 7.5% or 10% polyacrylamide gels followed by immunoblotting.

### siRNA Experiments

A nonspecific control duplex, synthetic siRNA duplexes against UbcH5a, and UbcH6 (IDT), and synthetic siRNA duplexes against UbcH7 (B-Bridge International) were purchased. The endoribonuclease-prepared siRNA duplexes against UbcH5c were synthesized as described previously [41]. Briefly, the RNA strand for the full-length coding region of *UbcH5c* was synthesized using the MEGAscript kit (Ambion) from PCR-derived linear templates carrying a phage T7 promoter at both ends. The dsRNA was digested by recombinant RNase III in 100 µl digestion buffer (20 mM Tris-HCl pH 7.9, 140 mM NaCl, 2.7 mM KCl, 5 mM MgCl_2_, 0.5 mM EDTA, 5% glycerol, 1 mM DTT) for 3 h at 37°C. The reaction was terminated by 20 mM EDTA and the products were separated on a 12% polyacrylamide gel in TBE. Both 21- and 27-bp DNA fragments were used to estimate the migration of RNA duplexes. Short RNAs of appropriate sizes were eluted from gel slices by soaking in 1 M (NH_4_)_2_AC at 37°C overnight and recovered by ethanol precipitation. The precipitate was dissolved in 30 µl of siRNA buffer (10 mM Tris-HCl pH 7.4, 0.5 mM EDTA).

To achieve a sufficient down-modulation of the UbcH5 isoforms, UbcH6, and UbcH7, 293 T cells were transfected with 80 µM siRNAs by using X-tremeGENE siRNA Transfection Reagent (Roche) according to the manufacturer’s protocol. Twenty-four hours after the siRNA transfection, the Myc-TdT and His-Ub plasmids were transfected by using GeneJuice Transfection Reagent (Novagen). After another 24 h incubation, MG132 treatment (final concentration, 10 µM) was performed for 12 h, and the cell lysates were subjected to SDS-PAGE and analyzed by immunoblotting.

### Real-time PCR

Total RNA was extracted using an RNAspin Mini (GE Healthcare) according to the manufacturer’s instructions, including an on-column DNase treatment step. RNA (2 µg) was incubated with random hexamers and a SuperScript VILO cDNA Synthesis Kit (Invitrogen) to generate cDNA. A twenty-fold dilution of the resulting cDNA was used for real-time PCR. Real-time PCR was performed with Thunderbird qPCR Mix (Toyobo) in quadruplicate using an ABI Prism 7300. Primers were as follows: GAPDH primers: 5′-GCACCGTCAAGGCTGAGAAC-3′, 5′-TGTGAAGACGCGCCAGTGGA-3′; UbcH5a primers: 5′-CGATCCACCTGCTCACTGTT-3′, 5′-TGAGAAAGAAGACTCCACCTTG-3′; UbcH5b primers: 5′-ATTGAATGATCTGGCACGGG-3′, 5′-TGTCATTTGGCCCCATTATTG-3′; UbcH5c primers: 5′-AGAGTGAGGAGCCAGACGACA-3′, 5′-CTGCAGAACATTGTGCTGCTGGAG-3′.

### DNA Binding Assay

His-TdT (180 ng) was incubated at 37°C for 1 h in the reaction *in vitro* ubiquitylation mixture with 20 pmol of biotinylated ssDNA coupled with 7 µl of streptavidin–agarose (Sigma). After stopping the reaction by adding 1.5 µl of 0.5 M EDTA, the beads were then washed three times with wash buffer C (50 mM Tris-HCl pH 7.4, 5 mM MgCl_2_, 2 mM NaF, 1 mM DTT, 0.02% Triton X-100) and boiled with Laemmli buffer. The eluates were subjected to SDS-PAGE followed by immunoblotting.

The binding of ubiquitylated Flag-TdT to ssDNA was carried out as follows. Purified poly-ubiquitylated Flag-TdT (200 ng) was incubated with 20 pmol of biotinylated ssDNA coupled with 7 µl of streptavidin–agarose (Sigma) in binding buffer B (50 mM Tris–HCl pH 7.4, 100 mM NaCl, 2.5 mM MgCl_2_, 10% glycerol, 0.1% TritonX-100, 200 µg/ml BSA) for 60 min at 4°C. After being washed with binding buffer B, the bound proteins were eluted with Laemmli buffer. The eluates were analyzed by SDS-PAGE and immunoblotting, using an anti-Flag antibody.

### Primer Extension Assay

To analyze the primer extension activity of ubiquitylated TdT, an *in vitro* ubiquitylation reaction was performed against Glutathione Sepharose 4B-bound GST-TdT with or without ATP. After an incubation for the specified time periods, the reactions were stopped by washing the beads twice with the reaction mixture for the primer extension assay, which contained 50 mM Tris-HCl (pH 7.5), 1 mM MnCl_2_, 1 mM DTT, 4% glycerol, 0.2 µM Cy5-labeled oligo(dT)20, and 0.1 mg/ml BSA. A reaction mixture with 0.1 mM dTTP was added to the beads. After incubation with constant agitation for 1 h at 30°C, reactions were stopped by adding 1/10 volume of 0.5 M EDTA and 4 M NaCl. The reaction mixture was removed from the pelleted beads and analyzed by 7 M urea/8% PAGE. Fluorescent bands were visualized on a Typhoon 9200 Gel Imager (GE Healthcare). Proteins were eluted from the beads with Laemmli buffer. Ubiquitylation of GST-TdT in the eluates was analyzed by SDS-PAGE and immunoblotting using an anti-TdT antibody.

## Supporting Information

Figure S1 **Inhibition of TdT ubiquitylation in E2-depleted cells.** 293 T cells in 6-well plates were transfected with a control siRNA or siRNAs targeting UbcH5c (20 nM), UbcH6 (50 nM) and UbcH7 (40 nM) alone or in combination with Myc-TdT (0.4 µg) using MultiFectam (Promega) 24 h prior to His-Ub transfection. The cells were then transfected with His-Ub (2.0 µg) using the X-tremeGENE HP DNA transfection reagent (Roche). After incubation for 24 h, the cells were treated with 10 µM MG132 for another 6 h. Ubiquitylated Myc-TdT, UbcH5, UbcH6, UbcH7, and actin in the lysate were detected by immunoblotting with an anti-Myc, anti-UbcH5, anti-UbcH6, anti-UbcH7, or anti-actin antibody. The ratio of mono-ubiquitylated Myc-TdT to unmodified Myc-TdT was determined with ImageJ.
(TIF)

Figure S2 **TdT ubiquitylation inhibits its nucleotidyltransferase activity.** (A) Purified unmodified, oligo-ubiquitylated, and poly-ubiquitylated Flag-TdT. 293 T cells were co-transfected with expression vectors encoding Flag-TdT (lanes 1–3) and His-Ub (lanes 2 and 3). Flag-TdT was purified by ANTI-FLAG M2 affinity gel (lane 1). Oligo- and poly-ubiquitylated Flag-TdT (Flag-TdT-Ub and Flag-TdT-Ubn, respectively) were purified using Ni Sepharose 6 Fast Flow and ANTI-FLAG M2 affinity gel (lanes 2 and 3, respectively). (B) Ubiquitylated TdT purified from 293 T cells binds to ssDNA. Poly-ubiquitylated Flag-TdT (200 ng) was incubated with (lane 2) or without (lane 3) biotinated ssDNA coupled with streptavidin–agarose. The proteins bound to DNA were subjected to immunoblot analysis using an anti-Flag antibody. (C) Primer extension assay. TdT activity was assayed by extension of biotinylated 34-mer ssDNA using Flag-TdT (lanes 3–5), Flag-TdT-Ub (lanes 6–8), or Flag-TdT-Ubn (lanes 9–11). Biotinylated 34-mer ssDNA was incubated in the reaction mixture together with 6 ng (lanes 3, 6, and 9), 20 ng (lanes 4, 7, and 10), or 60 ng (lanes 5, 8, and 11) of purified Flag-TdT, respectively. As a positive control, 50 ng of His-TdT was used in the reaction (lane 1). After electrophoresis by a 20% polyacrylamide gel, biotinylated ssDNA was transferred to Hybond N+ membrane and then cross-linked for 5 min by a UV trans-illuminator equipped with 312 nm bulbs. After blocking, biotinylated ssDNA was detected by a Streptavidin-HRP.
(TIF)
