# Supplementary figures and images for: Ubiquitylation of Terminal Deoxynucleotidyltransferase Inhibits Its Activity
Source: PLoS One. 2012 Jul 11;7(7):e39511. doi: 10.1371/journal.pone.0039511 (PMC3394778; doi:10.1371/journal.pone.0039511)

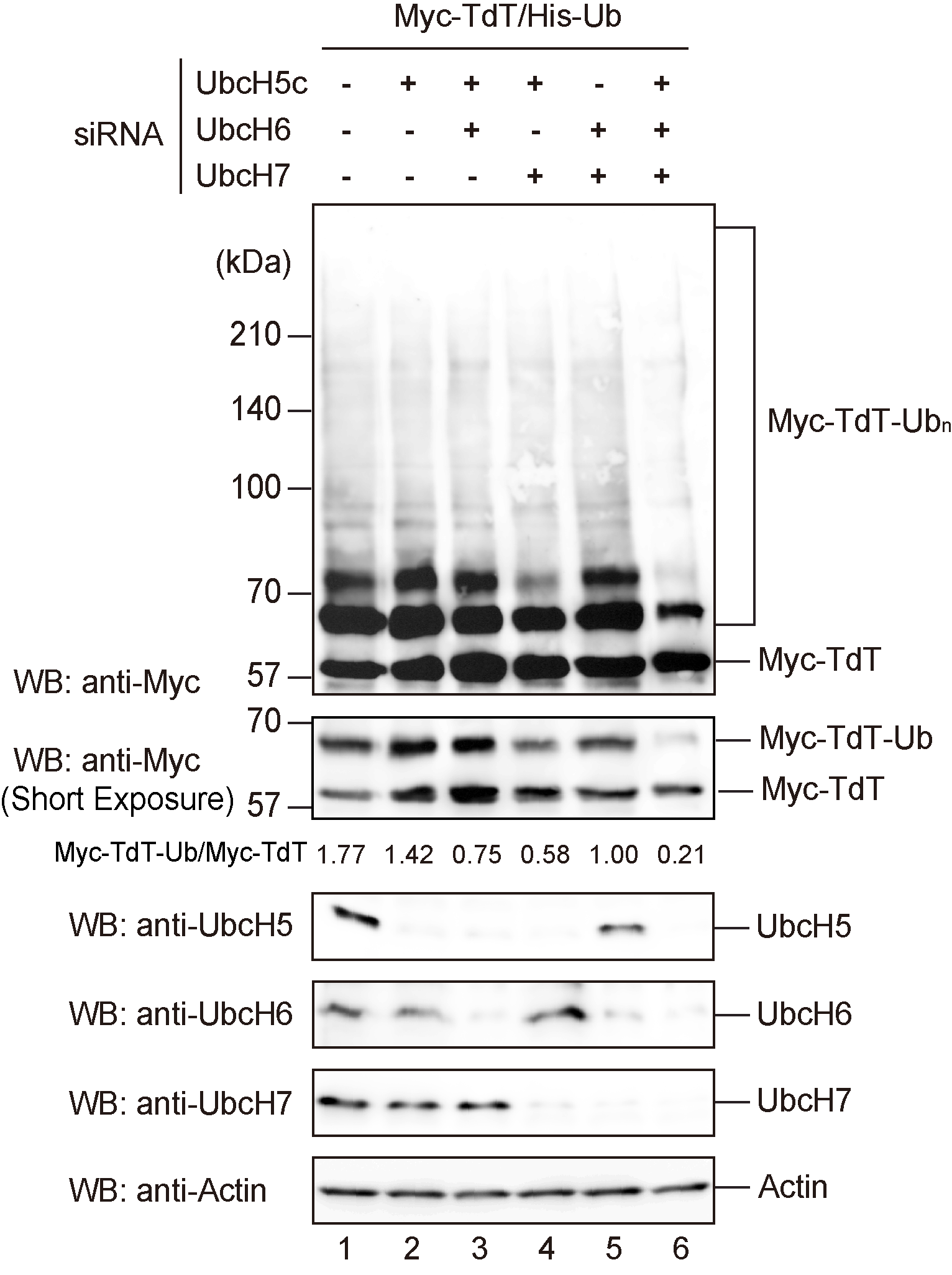

Supplement: Figure S1 — Inhibition of TdT ubiquitylation in E2-depleted cells. 293 T cells in 6-well plates were transfected with a control siRNA or siRNAs targeting UbcH5c (20 nM), UbcH6 (50 nM) and UbcH7 (40 nM) alone or in combination with Myc-TdT (0.4 µg) using MultiFectam (Promega) 24 h prior to His-Ub transfection. The cells were then transfected with His-Ub (2.0 µg) using the X-tremeGENE HP DNA transfection reagent (Roche). After incubation for 24 h, the cells were treated with 10 µM MG132 for another 6 h. Ubiquitylated Myc-TdT, UbcH5, UbcH6, UbcH7, and actin in the lysate were detected by immunoblotting with an anti-Myc, anti-UbcH5, anti-UbcH6, anti-UbcH7, or anti-actin antibody. The ratio of mono-ubiquitylated Myc-TdT to unmodified Myc-TdT was determined with ImageJ. (TIF) [file pone.0039511.s001.tif]

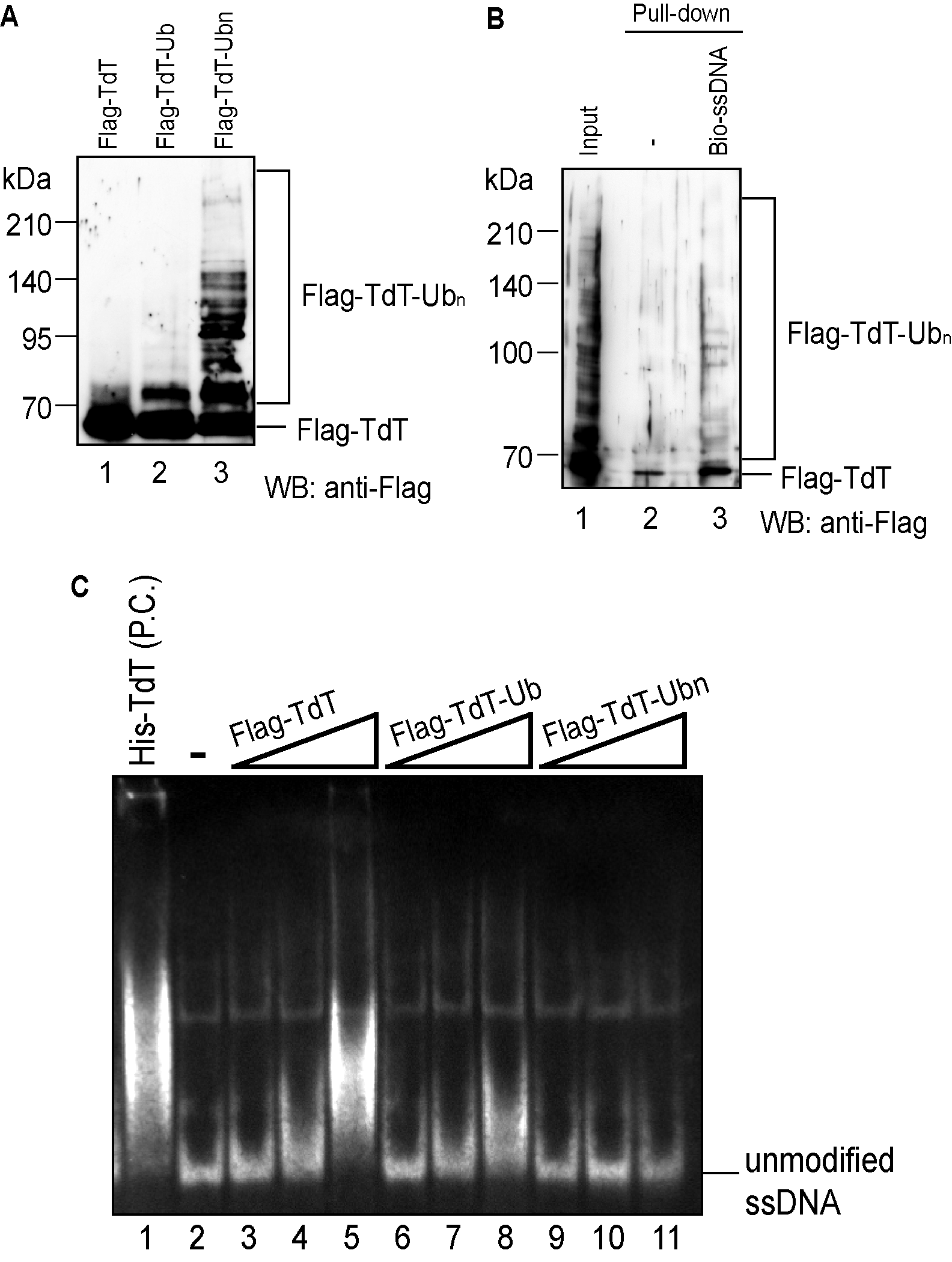

Supplement: Figure S2 — TdT ubiquitylation inhibits its nucleotidyltransferase activity. (A) Purified unmodified, oligo-ubiquitylated, and poly-ubiquitylated Flag-TdT. 293 T cells were co-transfected with expression vectors encoding Flag-TdT (lanes 1–3) and His-Ub (lanes 2 and 3). Flag-TdT was purified by ANTI-FLAG M2 affinity gel (lane 1). Oligo- and poly-ubiquitylated Flag-TdT (Flag-TdT-Ub and Flag-TdT-Ubn, respectively) were purified using Ni Sepharose 6 Fast Flow and ANTI-FLAG M2 affinity gel (lanes 2 and 3, respectively). (B) Ubiquitylated TdT purified from 293 T cells binds to ssDNA. Poly-ubiquitylated Flag-TdT (200 ng) was incubated with (lane 2) or without (lane 3) biotinated ssDNA coupled with streptavidin–agarose. The proteins bound to DNA were subjected to immunoblot analysis using an anti-Flag antibody. (C) Primer extension assay. TdT activity was assayed by extension of biotinylated 34-mer ssDNA using Flag-TdT (lanes 3–5), Flag-TdT-Ub (lanes 6–8), or Flag-TdT-Ubn (lanes 9–11). Biotinylated 34-mer ssDNA was incubated in the reaction mixture together with 6 ng (lanes 3, 6, and 9), 20 ng (lanes 4, 7, and 10), or 60 ng (lanes 5, 8, and 11) of purified Flag-TdT, respectively. As a positive control, 50 ng of His-TdT was used in the reaction (lane 1). After electrophoresis by a 20% polyacrylamide gel, biotinylated ssDNA was transferred to Hybond N+ membrane and then cross-linked for 5 min by a UV trans-illuminator equipped with 312 nm bulbs. After blocking, biotinylated ssDNA was detected by a Streptavidin-HRP. (TIF) [file pone.0039511.s002.tif]
